# How dose sparing of cardiac structures correlates with in‐field heart volume and sternal displacement

**DOI:** 10.1120/jacmp.v17i6.6324

**Published:** 2016-11-08

**Authors:** Taeho Kim, Kelli Reardon, Daniel M. Trifiletti, Constance Geesey, Kaitlyn Sukovich, Edwin Crandley, Paul W. Read, Krishni Wijesooriya

**Affiliations:** ^1^ Radiation Oncology University of Virginia Health System Charlottesville VA USA; ^2^ Radiation Oncology Virginia Commonwealth University Richmond VA USA

**Keywords:** predicting dose to heart, left‐breast irradiation

## Abstract

Cardiac irradiation increases the risk of coronary artery disease in patients with left‐sided breast cancer. Techniques exist to reduce cardiac irradiation, but the optimum technique depends on individual patient anatomy and physiology. We investigated the correlation of delta heart volume in field (dHVIF) and sternal excursion with dose sparing in heart and left anterior descending artery (LAD) to develop quantitative predictive models for expected dose to heart and LAD. A treatment planning study was performed on 97 left‐breast cancer patients who underwent whole breast radiotherapy (prescription dose = 50 Gy) under deep inspiratory breath hold (DIBH). Two CT datasets, free breathing (FB) and DIBH, were utilized for treatment planning and for determination of the internal anatomy‐based DIBH amplitude. The mean heart and LAD dose were compared between FB and DIBH plans and dose to the heart and LAD as a function of dHVIF and sternal excursion were determined. The [Average (STD); Range] mean heart doses from free breathing and DIBH are [120.5(65.2); 28.9 ~ 393.8] cGy and [67.5(25.1); 19.7 ~ 145.6] cGy, respectively. The mean LAD doses from free breathing and DIBH are [571.0(582.2); 42.2 ~ 2332.2] cGy and [185.9(127.0); 41.2 ~ 898.4] cGy, respectively. The mean dose reductions with DIBH are [53.1(50.6); ‐15.4 ~ 295.1] cGy for the heart and [385.1(513.4); ‐0.6 ~ 2105.8] cGy for LAD. Percent mean dose reductions to the heart and LAD with DIBH are 44% (p < 0.0001) and 67% (p < 0.0001), respectively, compared to FB. The dHVIF mean dose reduction correlation is 8.1 cGy/cc for the heart and 81.6 cGy/cc for LAD (with linear trend and y intercept: 26.0 cGy for the heart, 109.1 cGy for LAD). DIBH amplitude using sternal position was [1.3(4.8); .38 ~2.5] cm. The DIBH amplitude mean dose reduction correlation is 14 cGy/cm for the heart and 212 cGy/cm for LAD (with linear trend with y intercept: 35.6 cGy for the heart, 102.4 cGy for LAD). The strong correlation of dose sparing to the heart and LAD with dHVIF and sternal excursion suggests that mean dose sparing to heart and LAD can be predicted with either dHVIF or sternal excursion equally well. The metrics proposed could be utilized to allow providers to determine the relative dosimetric benefits of different heart‐sparing techniques as early as time of consultation.

PACS number(s): 87.53.Tf

## I. INTRODUCTION

There is level one evidence from multiple phase III clinical trials that adjuvant whole‐breast radiotherapy can reduce recurrence and breast cancer death.^1^, ^2^, ^3^ However, long‐term follow‐up studies indicate that incidental irradiation of the heart during external beam radiotherapy can increase the risk of cardiovascular damage, potentially limiting the long‐term survival benefit of adjuvant radiotherapy.^2^, ^4^, ^5^ Recently, a population‐based analysis of radiation‐induced cardiac toxicity following treatment of left‐sided breast cancer suggested a 7.4% relative increase in major coronary events per 1 Gy increase in mean heart dose.^(6)^

There are a variety of techniques available to reduce irradiation of the heart and left anterior descending artery (LAD) from the tangential left breast fields, including voluntary deep inspiration breath‐hold (DIBH)^(7)^ active breathing control (ABC)^(8)^ and prone positioning.^(9)^ Additionally, with audio and/or visual coaching, patients can be trained to take a deeper breath‐hold.^(10)^ Currently, there are no validated criteria available for physicians to aid in the selection between these techniques and free breathing.

The DIBH method physically increases the separation between the heart/LAD and the target volume to reduce the irradiated heart volume without degrading planning target volume (PTV) coverage.^10^, ^11^, ^12^, ^13^, ^14^ In the voluntary DIBH technique, patients are instructed verbally and/or visually to maintain their respiratory position during treatment delivery.^(10)^ Using this technique, the respiratory tumor motion is reduced and the separation of the target volume from critical structures is increased. A number of previous studies reported the significant reduction of cardiac doses with DIBH.^9^, ^11^, ^12^ For example, the comparison study with 17 breast cancer patients by Korreman et al.^(13)^ showed that the median heart volume receiving more than 50% of the prescription dose was reduced from 19.2% with free breathing (FB) to 1.9% with DIBH when the mean chest wall position with DIBH was 12.6 mm higher from that of FB. McIntosh et al.^(14)^ reported that DIBH reduced the mean heart dose by 48% with a 20.7 mm mean external breath‐hold sternal displacement using the Varian real‐time position management system (RPM: Palo Alto, CA). In addition, Bruzzaniti et al.^(11)^ reported that the mean dose was reduced from 168 cGy to 124 cGy for the heart and from 901 cGy to 274 cGy for LAD with DIBH, while the minimum distance between the heart and the target volume was increased from 14.3 mm with FB to 26.2 mm with DIBH (11.9 mm separation).

A parameteric model for cardiac dosimetric sparing with DIBH has not been fully established in routine clinical practice.^12^, ^15^ For example, Nissen and Appelt^(12)^ reported the lack of correlation between age/breath‐hold volume and the heart dose from 319 breast cancer patients. Register et al.^(15)^ showed dosimetric sparing with DIBH has a good correlation with heart volume in field (HVIF) and mean heart dose from 64 breast cancer patients. Although useful, its main limitation is that the dosimetric results are unknown until after radiotherapy planning when changing the technique may require repeat simulation and delay of treatment. In addition to HVIF, the breath‐hold amplitude based on sternal displacement could be a good predictor of dosimetric sparing because the breath‐hold position in DIBH is shown to be highly reproducible. Interfraction internal anatomy based DIBH amplitude reproducibility was shown to be approximately 3 mm.^(14)^

It would be novel and informative to demonstrate a relationship between breath‐hold amplitude based on sternal displacement and the dosimetric sparing of heart and LAD. This could potentially serve to inform physicians on the magnitude of benefit of DIBH and ABC prior to simulation and planning. In this report, we investigated for the first time the functional form of dose sparing to the heart and LAD with delta HVIF (dHVIF: direct predictor) and sternal excursion (indirect predictor) as predictors, and provide a predictive model for the expected dose sparing of heart and LAD from dHVIF and sternal displacement.

## II. MATERIALS AND METHODS

The treatment plans of 97 consecutive left‐sided breast cancer patients treated at our institution were retrospectively reviewed on this institutional review board‐approved study.

### A. CT simulation, internal breath‐hold amplitude, and whole breast treatment planning

**Figure 1:**
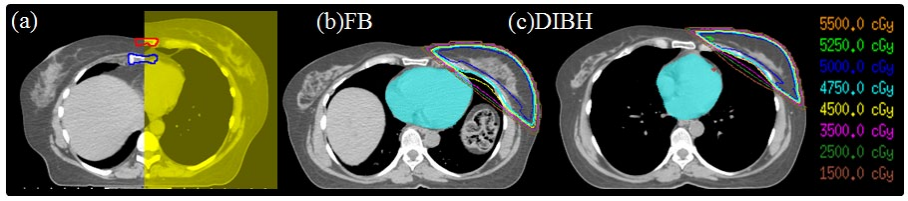
Anatomy‐based registration (a) of the two CT datasets, DIBH and FB. Contours of the sternum are shown: the red contour is on the DIBH dataset (yellow) and the blue one is on the FB dataset (gray). Isodose lines from plans on a sample patient on the (b) FB and (c) DIBH planning studies are shown. Organs at risk (OARs) were contoured (blue colorwash = heart,red colorwash = LAD).

Each patient had a free‐breathing and a DIBH CT scan. DIBH amplitude is defined as the difference between the center of the spinal cord and the sternum at the most anterior sternal position of the patient CT image. Sternal displacement is defined as the difference between the most anterior sternal position on the FB and DIBH datasets at the same axial position (based on vertebral body anatomy). The Varian RPM was used to monitor the breath‐hold amplitude during CT simulation, pretreatment imaging, and treatment delivery. Prior to the CT simulation, patients were instructed to take a deep inspiration and hold their breath. This was practiced while RPM system monitored and recorded the breath‐hold amplitude and the duration of the breath hold. CT simulation for this study proceeded only if the patient could hold their breath for more than 15 s. Typical CT scan time for both FB and DIBH took about 15 s, and were identical to a given patient's scan length. The sternal displacement was determined using the two CT datasets obtained at simulation. Anatomy‐based registration of the two CT datasets was performed using the Pinnacle treatment‐planning system (Philips Medical Systems, Fitchburg, WI), aligning rigidly based on vertebral body location. The sternum was contoured on each dataset. In Fig. 1, the sternum contour is displayed on the co‐registered free breathing (gray image with blue contour) and DIBH (yellow image with red contour) CT scans. The sternal displacement was determined by measuring the difference between the most anterior sternal position on the FB and DIBH datasets at the same axial position (based on vertebral body anatomy). The heart, LAD, lungs, PTV, and whole breast were contoured by a single experienced radiation oncologist on both datasets according to the RTOG Breast Cancer Contouring Atlas.^(16)^

Three‐dimensional conformal treatment plans were created on both CT datasets using the Pinnacle treatment planning system, each based on our institution's standard planning practice. Treatment plans were created with 6 or 15 MV tangential beams to cover all breast tissue, with a 2 cm flash on the anterior breast border and limiting the inclusion of lung to less than 2 cm. Medial and lateral nondivergent tangential fields were employed with a forward planned field‐in‐field technique to achieve adequate breast coverage and 95% of the breast volume was covered by the prescription dose. The treatment plans were modified until all breast tissue was covered with the 95% isodose line (DRX = 50 Gy, 2 Gy × 25 fractions). In Fig. 1, isodose lines of two treatment plans with (b) FB and (c) DIBH are presented visually demonstrating the reduction in heart irradiation with DIBH.

### B. Dosimetric analysis

The mean dose, max dose (RTOG volume of 0.03 cc to define the max dose), $V_{5}$, $V_{10}$, and $V_{30}$ to heart and LAD were assessed on the FB and DIBH plans (e.g., $V_{5}$: organ volume receiving 5 Gy or more). The heart volume within the 50% isodose line was defined as heart volume in field (HVIF). In addition, delta HVIF (dHVIF) was defined as the difference of HVIF between DIBH and FB.

### C. Correlation of dosimetric sparing with delta heart volume in field (dHVIF) and sternal excursion

Linear fitting of mean dose to the heart and LAD as a function of dHVIF and sternal displacement was performed to investigate the dosimetric sparing to heart and LAD with dHVIF and sternal displacement using the software, Origin (OriginLab, Northampton, MA).

### D. Statistical analysis

The significant absolute and relative difference of dose between FB and DIBH plans were quantified. Quantitative statistical comparison of mean dose to heart and LAD from the two different CT scans was performed using the paired Student's *t*‐test and evaluated in a spreadsheet program (Excel 2010, Microsoft, Redmond, VA). A p‐value of < 0.05 was considered statistically significant.

## III. RESULTS

Brief analyses are:

1. A very large range of breast volumes (change of order of magnitude) were included in this analysis: mean (STD) of breast volume = 1062.7(520.8), range = 242‐3051 cc.
2. There was no correlation between breast volume and the thoracic vertebral body level of the central slice of DIBH breast.
3. Mean (STD) of thoracic vertebral body level of mid plane of DIBH breast = 6.9(0.71), range = 5‐9.

### A. Internal breath‐hold amplitude

**Figure 2:**
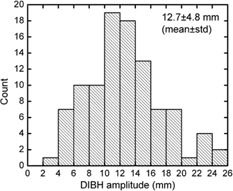
Histogram of internal DIBH amplitude for all patients is shown.

Figure 1 shows that the diaphragm of the patient is pushed downward, heart is elongated, moves away from the breast tangents in all three dimensions, rib cage is expanded with more air moving the sternum away from the spinal cord during DIBH. The mean DIBH amplitude using internal anatomy was 12.7 ± 4.8 mm. The median DIBH amplitude was 11.9 mm with a range of 3.8–25.1 mm. In Fig. 2, the histogram of DIBH amplitude is presented.

### B. Dosimetric analysis

Dosimetric values for the heart and LAD are presented in Table 1. Average reductions of the mean heart dose from 120.5 ± 65.2 cGy with FB to 67.5 ± 25.1 cGy with DIBH (44% reduction, p < 0.0001) and average reduction of the mean LAD artery dose from 571.0 ± 582.2 cGy with FB to 185.9 ± 127.0 cGy with DIBH (67% reduction, p < 0.0001) was observed.

The dosimetry analysis in terms of $V_{5}$, $V_{10}$, $V_{30}$, HVIF, dHVIF, and total volume of heart and LAD plans are presented in Table 2. Average reductions of $V_{5}$ to heart from 14.45 ± 16.2 cc with FB to 1.7 ± 3.4 cc with DIBH (88% reduction, p < 0.0001) and average reduction of $V_{5}$ to LAD from 0.8 ± 0.9 cc with FB to 0.1 ± 0.3 cc with DIBH (82% reduction, p < 0.0001) was observed. The [Average (STD); Range] HVIF from FB and DIBH are [3.5(5.8); 0.0 ~ 30.0] cc and [0.1(0.4); 0.0 ~ 3.8] cc. Of note only two plans with DIBH included HVIF more than 1 cc, while 45 plans with FB included HVIF more than 1 cc.

**Table 1:** Comparison of treatment plan parameters to heart and LAD: [Average (STD)] cGy. $\text{Difference}\;=\;{\text{Dose}}_{‐\text{FB}}‐{\text{Dose}}_{‐\text{DIBH}}(\text{cGy})$ and reduction rate (%) are presented

*Parameter*	*FB (cGy)*	*DIBH (cGy)*	*Difference (cGy)*	*p‐value*
*Heart*				
Max	3140.9 (1630.8)	1010.3 (1008.5)	2130.6 (1352.2) (‐44%)	< 0.0001
Mean	120.5 (65.2)	67.5 (25.1)	53.1 (50.6) (‐44%)	< 0.0001
*LAD*				
Max	2064.4 (1735.3)	646.9 (738.2)	1417.5 (1399.8) (‐69%)	< 0.0001
Mean	571.0 (582.2)	185.9 (127.0)	385.1 (513.4) (‐67%)	< 0.0001

**Table 2:** Comparison of treatment plan parameters to heart and LAD: organ volume receiving radiation dose (cc). $\text{Difference}\;=\;{\text{Volume}}_{‐\text{FB}}‐{\text{Volume}}_{‐\text{DIBH}}(\text{cc})$ and reduction rate (%) are presented

*Parameter*	*FB (cc)*	*DIBH (cc)*	*Difference (cc)*	*p‐value*
*Heart*				
$V_{5}$	14.4 (16.2)	1.7 (3.4)	12.8 (14.3) (‐88%)	< 0.0001
$V_{10}$	7.3 (9.8)	0.4 (1.3)	6.9 (9.5) (‐95%)	< 0.0001
$V_{30}$	2.4 (4.5)	0.0 (0.2)	2.4 (4.4) (‐99%)	< 0.0001
HVIF	3.5 (5.8)	0.1 (0.4)	dHVIF: 3.4 (5.7) (‐98%)	< 0.0001
Total volume	605.2 (118.7)	553.3 (105.9)	51.9 (63.9)	
*LAD*				
$V_{5}$	0.8 (0.9)	0.1 (0.3)	0.6 (0.7) (‐82%)	< 0.0001
$V_{30}$	0.2 (0.4)	0.0 (0.0)	0.2 (0.4) (‐99%)	< 0.0001
Total volume	3.5 (1.3)	3.2 (1.1)	0.3 (0.8)	

### C. Correlation of dose sparing in heart and LAD with dHVIF and sternal displacement

#### C.1 Direct predictor

**Figure 3:**
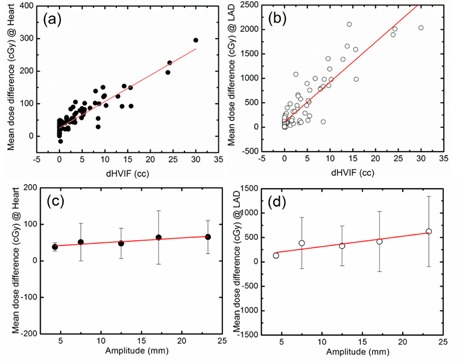
Correlation of the mean dose reduction to dHVIF: (a) mean dose to heart, (b) mean dose to LAD artery with dHVIF are presented. Correlation of the mean dose reduction to mean DIBH amplitude with 5 mm bin size. (c) Mean dose to heart, (d) mean dose to LAD artery with sternal excursion are presented. The red solid line is the linear fit to the measured data (y = Ax + B: (a)A=8.1,B=26.0cGy,R2=0.84,(b)A=81.6,B=109.1cGy,R2=0.82,(c)A=1.4,B=35.6cGy,R2=0.81,(d)A=21.2,B=102.4cGy,R2=0.81). It is noted that the linear fit to the measured data with B = 0 are $(a)A=10{.1,R}^{2}=0.65,(b)A=90{.2,R}^{2}=0.79,(c)A=3{.5,R}^{2}=‐1.77,(d)A=27{.4,R}^{2}=0.72$ (fitting lines not included in the figure).

Linear fitting of mean dose reductions as a function of the dHVIF was performed. Dose reduction as a function of the dHVIF changes are given by the formula: dose reduction (cGy) = 8.1 (cGy/cc) × dHVIF (cc) + 26.0 (cGy) for mean heart dose and dose reduction (cGy) = 81.6 (cGy/cc) × dHVIF (cc) + 109.1 (cGy) for mean LAD artery dose, as shown in Figs. 3(a) and (b). The red solid line is the linear fit to the measured data (scattered symbols: (a) filled circle – heart, (b) open circle – LAD artery). The mean dose reduction with dHVIF (3.4 cc) is 53.5 cGy for the heart and 386.5 cGy for LAD artery.

#### C.2 Indirect predictor

Linear fitting of mean dose reductions as a function of the sternal displacement is presented in Figs. 3(c) and (d). In contrast to dHVIF (direct predictor), linear fitting of mean dose reduction was performed with mean sternal displacement (5 mm bin size) because of large variation shown in Figs. 3(c) and (d) (error bars). Dose reduction as a function of the sternal displacement changes are given by the formula: dose reduction (cGy) =1.4(cGy/mm) × sternal displacement (mm) + 35.6 (cGy) for mean heart dose and dose reduction (cGy) = 21.2 (cGy/mm) × sternal displacement (mm) + 102.4 (cGy) for mean LAD artery dose. The red solid line is the linear fit to the measured data (scattered symbols: (c) filled circle – heart, (d) open circle – LAD artery). The mean dose reduction with mean sternal displacement (12.7 mm) is 48.4 cGy for the heart and 371.6 cGy for LAD artery. Predictive power of the following formula is only valid within the range (3.8–25.1 mm) shown in Figs. 3(c) and (d), where we have measurements.

It is noted that, the linear fit to the each measured data with one variable (B = 0) is $(a)A\;=\;10{.1,R}^{2}\;=\;0.65,(b)A\;=\;90{.2,R}^{2}\;=\;0.79,(c)A\;=\;3{.5,R}^{2}\;=\;‐1.77,(d)A\;=\;27{.4,R}^{2}\;=\;0.72$ (fitting lines not included in the figure). Since there can be many unknown reasons for the best linear fit to not go through the zero amplitude point, including a nonlinear behavior at very low amplitudes, we would not recommend extrapolation of this functional form with the zero linear fit. Hence it is given here only as a clarification.

## IV. DISCUSSION

In this study, we investigated the dose sparing to the heart and LAD with 3D conformal treatment plans using DIBH with dHVIF as a direct predictor and sternal displacement as an indirect predictor. Not surprisingly, we found that dHVIF has a strong correlation with cardiac dose reduction (less heart in the radiation field resulted in less heart dose). In addition, we found that sternal displacement also shows a correlation with cardiac dose reduction with 1.4 cGy/mm in mean heart dose and 21.2 cGy/mm in mean LAD dose.

In this study, the mean internal DIBH amplitude was 12.7 mm (range: 3.8–25.1 mm). However, in McIntosh et al.^(14)^ measurements showed that the breath‐hold position in DIBH is highly reproducible over the fractions and the mean breath‐hold variation using the internal anatomy land marks was 3 mm. Therefore DIBH treatments could deliver the planned dose accurately with minimum positional uncertainties. As shown in Fig. 1, physical separation between the heart/LAD and the target volume was increased by DIBH (sternal excursion: indirect predictor), resulting in decreased overlap between the heart and LAD and the radiation field (dHVIF: direct predictor) and the mean dose to heart and LAD was reduced in the DIBH plan.

Since the standard deviation of heart volumes was about 100 cc, this is well within the variation in Table 2. We suspect that the reason for this difference is that the heart changes shape with DIBH. It is also possible that pressure changes in the chest during DIBH affect the volume of the cardiac chambers, or the amount of time spent in different phases of the cardiac cycle. However, it is noteworthy that, since the FB heart volume is about 50 cc higher than the DIBH heart volume, mean dose to heart from DIBH compared to FB should be higher than what we report.

It is noted that the actual mean dose to heart is low with both techniques. For example, average dose to heart from 120.5 cGy with FB to 67.5 cGy with DIBH. Although the recently published population‐based analysis of radiation‐induced cardiac toxicity following treatment of breast cancer^(6)^ reported a dose response of the heart with a 7.4% relative increase in major coronary events per 1 Gy increase in mean heart dose, mean heart dose may not be the single most important quantity that relates to clinically detectable cardiac toxicity. Whether it is a combination of low‐dose bath, $V_{5}$ to heart, maximum dose to heart, or LAD, all parameters demonstrated significant reductions under DIBH. LAD is closer to the left breast than the heart and the mean dose reduction to LAD shows a steeper gradient with dHVIF and sternal displacement. Although the long‐term clinical significance of dose delivered to the LAD is largely unknown, it is nonetheless considered important because of the severe clinical implications of atherosclerosis of the LAD.

Despite mean dosimetric improvements with DIBH, there were select patients for whom DIBH provided relatively minor improvements, and it is possible that alternative treatment techniques, such as prone positioning or IMRT, could have benefitted these patients. Results from our data should be verified in terms of sensitivity and specificity to evaluate the possibility of using the sternal displacement as a predictor of the magnitude of benefit of DIBH.

There is the potential for clinicians to evaluate sternal excursion clinically and use this information to predict a potential benefit of DIBH in reducing the cardiac irradiation. We looked at the correlation of FB left lung volume vs. the breath‐hold amplitude hoping to use the diagnostic CT to predict the breath‐hold amplitude prior to CT sim at radiation therapy. However, there was no correlation between the FB left lung volume and the breath‐hold amplitude. In contrast, we found a slight correlation between the left lung volume difference (DIBH‐FB) and the breath‐hold amplitude. It is given by the linear relationship: $y\;=\;\text{Ax}+B,A\;=\;31.95,\;B\;=\;527.40,\;R^{2}\;=\;0.25$ (figure not shown).

**Figure 4:**
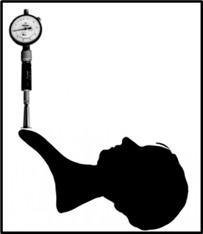
Sternal excursion measurements at first consult before CT simulation.

We propose that, during consultation and breast exam, clinicians evaluate patients in the general treatment position and ask them to take a deep breath and hold it. During this maneuver, clinicians can evaluate (and potentially measure) the sternal excursion clinically using a simple apparatus, as shown in Fig. 4. For example, sternal excursion can be measured using a digital linear measurement probe at first consult before CT simulation. According to our findings, the mean DIBH amplitude using internal anatomy was 12.7 mm. With 10 mm of the sternal displacement, the dose reduction will be 49.6 cGy in mean heart dose and 314.4 cGy in mean LAD artery dose, indicating a 3.7% relative decrease in major coronary events per 0.5 Gy decrease in mean heart dose based on the recently published population‐based analysis of radiation‐induced cardiac toxicity. However, predictive power of the formulas given in the result sections C1 and C2 is only valid within the sternal excursion range (3.8–25.1 mm), where we have measurements. In the future, it will be useful to have more data in the higher and lower breath‐hold displacement regions, so that we can have a more accurate prediction model of the heart/LAD dose.

If the excursion is greater than 1 cm, we propose that a DIBH scan alone is adequate without an additional FB scan. If, however, sternal excursion is minimal, a DIBH and FB should be performed at time of simulation to compare opposed tangential fields to other delivery techniques (i.e., planning and treatment purposes such as intensity‐modulated radiotherapy). A selected subset of patients could also be sent home with coaching instructions to help improve the sternal excursion prior to CT simulation, after the first consult, and the evaluation of sternal shift. Our institution is currently evaluating this proposed approach. An alternative approach is, prior to the CT scan, at the sim table, to measure the patient's sternal displacement. If the sternal displacement is < 1 cm (i.e., < 49.6 cGy reduction in mean heart dose), benefits of DIBH may not be substantial; so alternative treatments, such as a prone position or IMRT, can be considered. For clinical implementation, it is important to further evaluate the sensitivity and the specificity of this technique by performing a prospective clinical study.

## V. CONCLUSIONS

DIBH significantly spares dose to LAD and heart. The correlation between sternal excursion and dosimetric sparing may possibly suggest that mean dose sparing to LAD, and heart linearly increases with dHVIF and sternal excursion. These findings can serve to inform and improve clinical practice during the treatment technique selection process.

## COPYRIGHT

This work is licensed under a Creative Commons Attribution 3.0 Unported License.
